# Coherence measurements of polaritons in thermal equilibrium reveal a power law for two-dimensional condensates

**DOI:** 10.1126/sciadv.adk6960

**Published:** 2024-05-03

**Authors:** Hassan Alnatah, Qi Yao, Jonathan Beaumariage, Shouvik Mukherjee, Man Chun Tam, Zbigniew Wasilewski, Ken West, Kirk Baldwin, Loren N. Pfeiffer, David W. Snoke

**Affiliations:** ^1^ Department of Physics, University of Pittsburgh, 3941 O’Hara Street, Pittsburgh, PA 15218, USA.; ^2^ Joint Quantum Institute, University of Maryland and National Institute of Standards and Technology, College Park, MD 20742, USA.; ^3^ Department of Electrical and Computer Engineering, University of Waterloo, Waterloo, ON, Canada.; ^4^ Waterloo Institute for Nanotechnology, University of Waterloo, Waterloo, ON, Canada.; ^5^ Department of Electrical Engineering, Princeton University, Princeton, NJ 08544, USA.

## Abstract

We have created a spatially homogeneous polariton condensate in thermal equilibrium, up to very high condensate fraction. Under these conditions, we have measured the coherence as a function of momentum and determined the total coherent fraction of this boson system from very low density up to density well above the condensation transition. These measurements reveal a consistent power law for the coherent fraction as a function of the total density over nearly three orders of its magnitude. The same power law is seen in numerical simulations solving the two-dimensional Gross-Pitaevskii equation for the equilibrium coherence.

## Teaser

High-resolution measurements of the coherence of a homogeneous, thermal two-dimensional Bose gas reveal a universal power law.

## INTRODUCTION

Bose-Einstein condensation (BEC) is a remarkable state of matter in which a macroscopically large number of bosons act as a single, coherent wave. The physics of two-dimensional (2D) BEC has subtle differences from the three-dimensional case because thermal fluctuations destroy the long-range order in systems of reduced dimensions (*1*). However, a quasicondensate state can exist with strongly correlated coherence over finite distances, as predicted by Berezinskii (*2*) and Kosterlitz and Thouless (*3*). Microcavity exciton-polaritons (called here simply “polaritons”) are good candidates for the investigation of 2D boson systems because they allow direct experimental accessibility to the coherence in situ without destructive measurements. In most experiments with cold atoms that have tried to establish a phase diagram, only the momentum distribution or spatial profile has been measured, not the coherence directly (*4*–*7*).

Polaritons can be viewed as photons dressed with an effective mass and repulsive interactions, due to the strong coupling of a cavity photon state and a semiconductor exciton state. These particles have been shown to demonstrate Bose condensation and coherent effects in various experiments for nearly two decades [e.g., (*8*–*13*)]. Although, in many experiments, the polaritons have fairly short lifetime, leading to nonequilibrium condensates, in the past 10 years, microcavity structures have been available with polariton lifetime of several hundred picoseconds (*14*, *15*), which has allowed demonstration of true equilibrium, as seen in near-perfect fits to an equilibrium Bose-Einstein energy distribution up to the Bose-degenerate regime (*16*) and in a thermal power law of the spatial correlation (*17*).

Sun *et al.* (*16*) showed equilibrium in the degenerate regime up to five to six particles in the ground state, but, at higher densities, the occupation-number distribution *N*(*E*) deviated from a purely equilibrium distribution. We have since established that this was primarily due to the condensate becoming spatially inhomogeneous. In this work, we report experiments in which equilibrium is well established in a homogeneous polariton gas well up to ground-state occupations in the range of 100 to 1000. Although the particles have a lifetime for decay that is replenished by a steady-state pump, the lifetime of the particles is long compared to their thermalization time, so that only a tiny fraction of the population is lost and replaced at any point in time.

This allows us to perform accurate measurements of the coherence of the gas over a wide range of density. Because the gas is thermal and homogeneous, it allows direct comparison to theories for the coherence of a Bose gas in 2D. Although this type of experiment has been attempted with cold atoms (*18*), interference measurements in a cold atom gas are intrinsically a destructive measurement, and those measurements had low resolution.

These experiments can be interpreted as measuring the “condensate fraction” of the system, but, in a 2D system, the definition of the condensate fraction is somewhat controversial. Several theoretical papers [e.g., (*19*, *20*)] have defined the “condensate” as only those particles with strictly zero momentum. These theories kept no track of the phase coherence, only the populations of *k*-states. However, the crucial aspect of the Gross-Pitaevskii equation, which allows superfluid behavior such as quantized vorticity, is the phase coherence, and the Gross-Pitaevskii equation makes no sharp distinction between the Fourier components of a coherent wave with *k* = 0 and Fourier components with nonzero *k*. In addition, if the condensate is defined as only particles with strictly zero momentum, then the condensate fraction in 2D has vanishingly small value in the thermodynamic limit; this is an unhelpful definition for a finite system because it is well known (*1*) that a 2D system can have coherence on finite length scales. Instead, one can define the “coherent fraction” as the fraction of the particles that have 100% fringe visibility in an interference measurement, which is equivalent to the integral of the fringe visibility over the total set of momentum states. This is well-defined for any finite area. This may be termed the “quasicondensate,” because it corresponds to that part of the gas that has a single-valued wave function that obeys the Gross-Pitaevskii equation. Some, however, may restrict the term quasicondensate to a state with long-range, power-law correlation (*21*), while, at low density, there is exponential decay of the correlation of the coherence (as seen in our numerical model and presented in the Supplementary Materials). What we see experimentally is that there is no sharp cutoff between the coherent fraction at low density and the quasicondensate at high density—the coherence increases continuously from very low density up to near 100% at high density. Here, we will use the term coherent fraction to avoid confusion.

In this work, we undertake a detailed experimental and theoretical investigation of coherence as a function of the polariton gas density and determine the coherent fraction as a function of total particle density. First, we establish the polariton gas is in thermal equilibrium. We then determine the coherent fraction and compare it to numerical solutions of a 2D Gross-Pitaevskii equation.

## RESULTS AND DISCUSSION

The sample was cooled in a continuous-flow cold-finger cryostat at ∼5 K and excited nonresonantly with a continuous-wave laser, which was modulated by an optical chopper at 404 Hz with a duty cycle of 1.7% to prevent sample heating. The pump profile was shaped into a broad Gaussian with full width at half maximum of ∼65 μm. The nonresonant excitation created a plasma of electrons and holes, which spontaneously form excitons. These hot excitons then scatter down in energy to become polaritons.

**Fig. 1.**
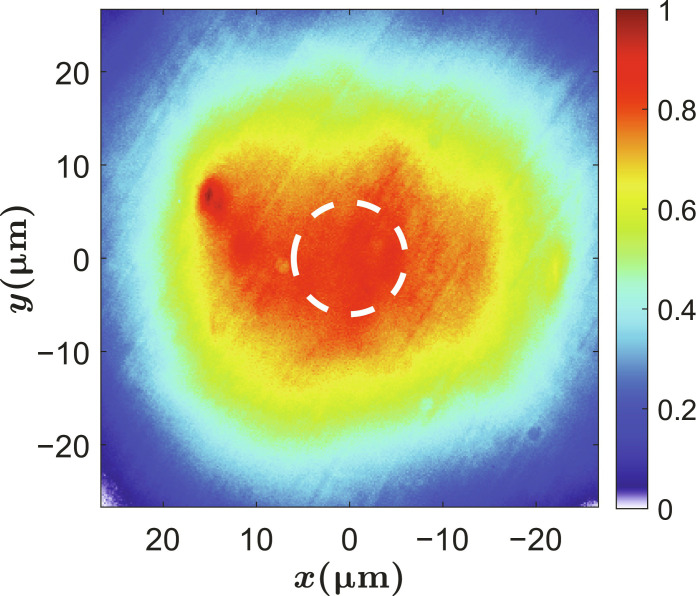
Real-space polariton emission. Polariton emission created by a wide area nonresonant pump. The white dashed circle indicates the region where the photoluminescence (PL) is collected.

The cavity detuning was δ = 2.5 meV, corresponding to an exciton fraction ∣*X*∣^2^ = 0.55 for the lower polariton at *k* = 0. The photoluminescence (PL) was collected using a microscope objective with a numerical aperture of 0.75 and was imaged onto the entrance slit of a spectrometer. The image was then sent through the spectrometer to a charge-coupled device for time-integrated imaging. A spatial filter was placed at the real-space plane to collect PL from a region where the gas was very homogeneous (typical diameter of 12 μm, as shown by the white dashed circle in Fig. 1).

**Fig. 2.**
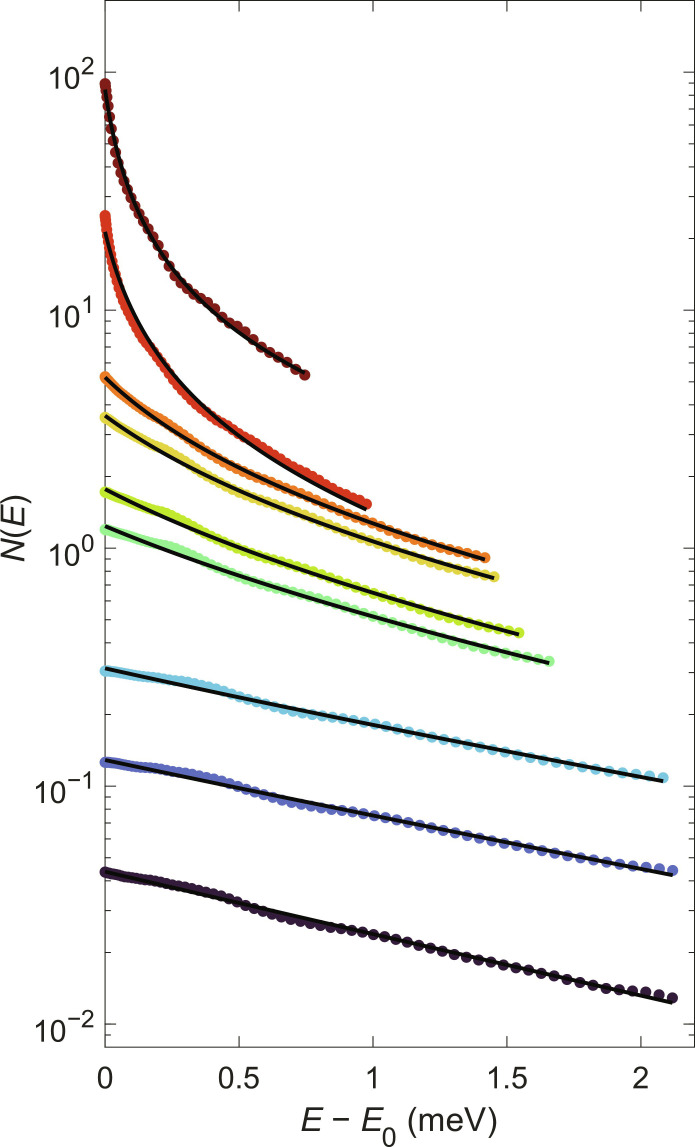
Equilibrium distribution of polaritons. Occupation of the lower polariton as a function of energy. The solid lines are best fits to the equilibrium Bose-Einstein distribution in Eq. 1. The temperature and chemical potential extracted from the fit are shown in Fig. 3. The power values from low to high are 0.008, 0.031, 0.132, 0.530, 0.653, 0.821, 0.940, 1.164, and 1.265 times the threshold pump power. The threshold power *P*_th_ is defined in the Supplementary Materials.

**Fig. 3.**
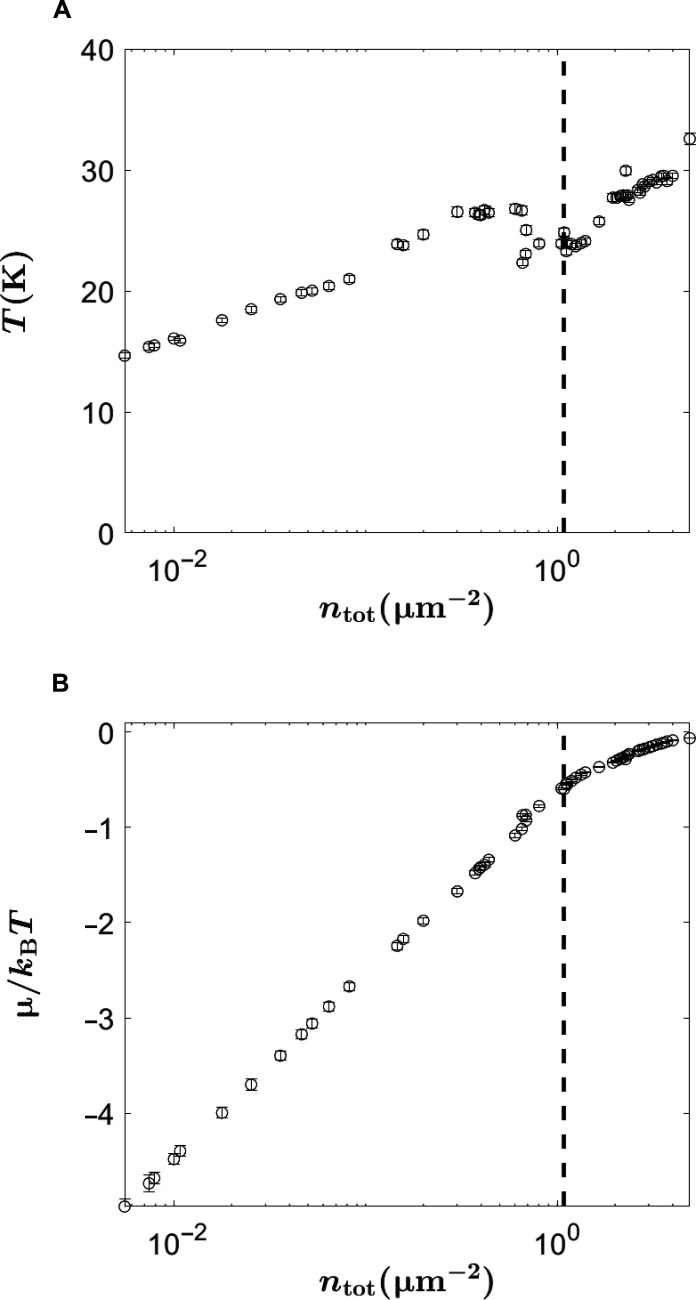
Extracted temperature and chemical potential. (**A**) The effective temperature of the polariton gas and (**B**) the reduced chemical potential obtained from the fits to the Bose-Einstein distribution. The vertical dashed line denotes when the occupation at *E* = 0 becomes equal to one, i.e., *N*(*E* = 0) = 1.

To show that polaritons can achieve thermal equilibrium, we measured the lower polariton occupation and we compared it to occupation number predicted by Bose-Einstein statistics. The lower-polariton occupation was measured by angle-resolved imaging, giving the intensity *I*(*k*, *E*), which is then converted to an occupation number *N*(*E*) using a single efficiency factor (the calibration of this factor is discussed in the Supplementary Materials). The measured polariton occupation for different pump power values is shown in Fig. 2. The measured occupation numbers were fit to a Bose-Einstein distribution, given by

$$N\left(E_{\text{LP}}\right)=\frac{1}{e^{\left[E_{\text{LP}}-E_{\text{LP}}\left(0\right)-\mu \right]/k_{B}T}-1}$$ (1)

where *T* and μ are the temperature and chemical potential of the polariton gas, respectively; *E*_LP_ is the lower polariton energy; and *E*_LP_(0) is the polariton ground state energy at *k* = 0, which shifts to higher energy as the density increases, due to many-body renormalization (*16*). The fits to Bose-Einstein distribution were done by using *T* and μ as fit parameters. As seen in Fig. 2, the experimental polariton occupation is well described by a Bose-Einstein distribution for all densities, indicating that the polariton gas is in true thermodynamic equilibrium. At densities well below the condensation threshold, the Bose-Einstein distribution becomes a Maxwell-Boltzmann distribution *N*(*E*_LP_) ∼ *e*^μ/*k*_B_T^*e*^−*E*_LP_/*k*_B_*T*^, which corresponds to a straight line on a semilog plot. However, when quantum statistics become important [i.e., *N*(*E*_LP_) ∼ 1], the shape of the distribution changes and an upturn at in low-energy states appears. The temperature and the chemical potential obtained from the fit to Bose-Einstein distribution are shown in Fig. 3. We emphasize that a single efficiency factor is used for all the distributions and only *T* and μ were varied.

**Fig. 4.**
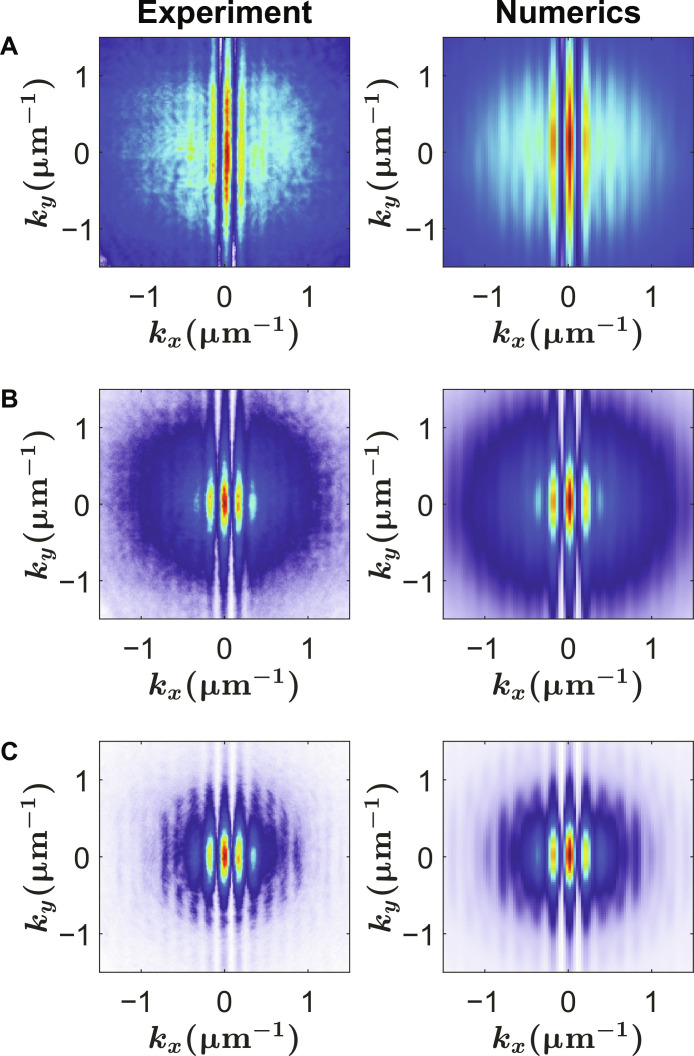
Interference pattern. The interference pattern in *k*-space obtained from the experiment (left column) and the numerics (right column) for three different densities, (**A**) *n* = 1.5 μm^−2^, (**B**) *n* = 4.5 μm^−2^, and (**C**) *n* = 9.3 μm^−2^.

The coherent fraction was measured by interfering the light emitted by the polariton gas **E**(k_x_, k_y_, t_0_ with its mirror symmetric image **E**(−k_x_, k_y_, t_0_) using Michelson interferometry. The resulting intensity pattern exhibits interference fringes, indicating the emergence of extended coherence. A typical interference pattern in *k*-space is shown in Fig. 4 for different pump powers. We use these interference patterns to extract coherent fraction of the polariton gas as the fringe contrast gives a direct measurement of the level of coherence.

To extract the coherent fraction, we assume that interference pattern is described by a partially coherent wave with a momentum-dependent amplitude *N*(*k*)

$$I\left(k\right)=N\left(k\right)\left[1+\alpha e^{-k/\kappa }\text{cos}\left(\lambda k\right)\right]$$ (2)

where κ is a fit parameter giving the region of coherence, α is a fit parameter ranging between 0 and 1 giving the degree of coherence, and λ is the component associated with the fringe spacing. Therefore, the coherent fraction can be defined as

$$\frac{n_{0}}{n_{\mathrm{tot}}}=\frac{\alpha \int ‍d^{2}k\;N\left(k\right)e^{-k/\kappa }}{\int ‍d^{2}k\;N\left(k\right)}$$ (3)

In the limit κ → ∞, Eq. 2 reduces to the interference pattern for fully coherent classical waves and the coherent fraction *n*_0_/*n*_tot_ → 1.

**Fig. 5.**
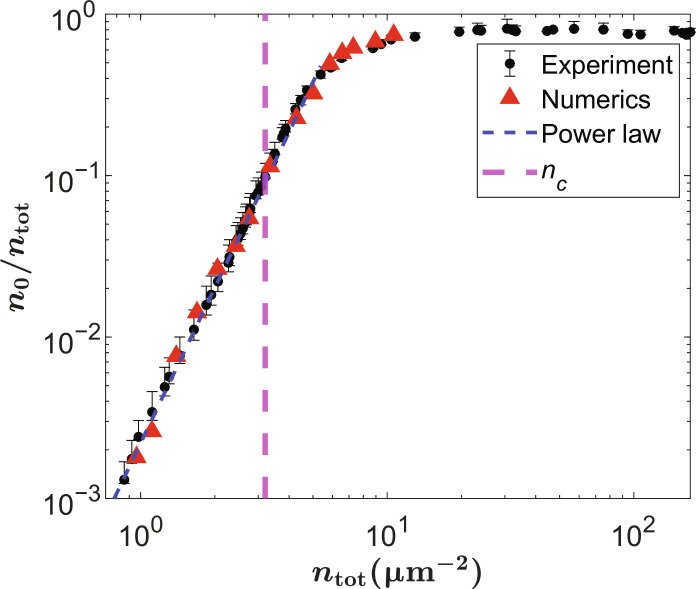
Coherent fraction. Black circles: Experimentally measured coherent fraction as a function of the total polariton density for a pinhole with an area *A* = π(6 μm)^2^. The quasicondensate fraction is defined in Eq. 3. Red triangles: Coherent fraction defined the same way, for the numerical simulations. Blue line: *n*^3.2^ power law. The vertical dashed line denotes the critical density, which is defined as the total density of polaritons at the threshold power *P*/*P*_th_ = 1, defined in the Supplementary Materials.

A notable result of these measurements is that the increase of the coherent fraction obeys a well-defined power law over a wide range of density, nearly three orders of its magnitude, as the density increases through the critical value. Figure 5 shows a typical dataset; as shown in the Supplementary Materials, many different datasets, including different values of the aperture size for the area of integration, can all be collapsed onto a single, universal curve. As discussed in the next section, this power law behavior is reproduced by a simple numerical solution of the Gross-Pitaevskii equation with no dissipation.

### Theory and numerical simulation

Because the experimental Bose gas is thermal and homogeneous, we can model the system using the Gross-Pitaevskii equation for the simplest case to get a universal result, which applies to any number-conserving, spatially homogeneous, 2D Bose gas in thermal equilibrium.

We solve the following Gross-Pitaevskii equation with noise introduced in the initial conditions

$$i\hbar \frac{\partial \psi \left(r,t\right)}{\partial t}=\left[-\frac{\hbar^{2}\nabla^{2}}{2m}+g_{c}|\psi \left(r,t\right){|}^{2}\right]\psi \left(r,t\right)$$ (4)

where *m* is the mass of the polaritons and *g_c_* is the repulsive polariton-polariton interaction. Significantly, we do not include any terms for generation or decay of the polaritons, because, as discussed above, the lifetime of the polaritons is long enough that these can be taken as negligible for the relevant dynamics, so that the system can be treated as number-conserving and in equilibrium.

To eliminate a computationally expensive transient regime, we start the system in an incoherent equilibrium state

$$\begin{array}{c} \psi \left(x,y,t=0\right)=\sum_{k_{n}}‍\sum_{k_{m}}‍\sqrt{N\left(\sqrt{k_{n}^{2}+k_{m}^{2}}\right)}\;e^{i\left(k_{n}x+k_{m}y\right)}\\ \times e^{i\left(\theta_{k_{n}}+\theta_{k_{m}}\right)} \end{array}$$ (5)

where *N*(*k*) = {*e*^[*E*(*k*) − μ]/*k*_B_*T*^ − 1}^−1^ is the Bose-Einstein distribution and *E*(*k*) = *ℏ*^2^*k*^2^/2*m*. The phases θ*_k_n__* and θ*_k_m__* are random numbers that are uniformly distributed in the interval [0,2π]. The system is then evolved in time until the system reaches a constant degree of coherence. To calculate the coherent fraction from the simulations, we first compute the interference pattern between *k_x_* and −*k_x_*

$$\begin{array}{c} I\left(k_{x},k_{y}\right)=\frac{1}{t_{\text{max}}}\int_{0}^{t_{\text{max}}}‍dt\;|\psi \left(k_{x},k_{y},t\right)e^{ik_{x}x_{0}}\\ +\psi \left(-k_{x},k_{y},t\right)e^{-ik_{x}x_{0}}{|}^{2} \end{array}$$ (6)

where *x*_0_ is a constant that defines the fringe spacing and *t*_max_ is the total simulated time. ψ(*k_x_*, *k_y_*, *t*) is the Fourier transform of the wave function in real space. Because in the experiment we use a pinhole to only collect light from an area *A* = π*r*^2^, we apply the same kind of filtering in the numerics for the real space wave function ψ(*x*, *y*, *t*) before calculating the Fourier transform ψ(*k_x_*, *k_y_*, *t*). The interference pattern *I*(*k_x_*, *k_y_*) is evaluated by averaging over several independent stochastic paths for each random initial condition as described by Eq. 5. Figure 4 shows a comparison between the experimentally measured interference pattern and the results obtained from the theoretical modeling, showing a very good agreement for the fringe visibility for different densities.

The coherent fraction from the numerical simulations is then calculated by following the same fitting procedure that was described in the previous section, namely, Eqs. 2 and 3 (for more details, see the Supplementary Materials). In both the experiment and the numerics, we subtracted the coherent fraction found in the zero-density limit, which corresponds to the coherence due to instrumental response, seen even in the Maxwell-Boltzmann limit. As seen in Fig. 5, our experimentally measured and numerically calculated coherent fraction show a very good agreement, with the same *n*^3.2±0.12^ power law over nearly three orders of magnitude of the value of the coherent fraction. At the highest densities, the coherent fraction of course cannot exceed unity and, therefore, saturates.

As discussed in the Supplementary Materials, the numerical model also gives us the in-plane coherence length of the gas as a function of density, which gives the same power law of 3.2 when converted to an area. In general, the numerics allow us to explore a wide range of conditions that agree with the experiments in all of the areas where we can compare them.

The agreement with the Gross-Pitaevskii numerical simulations for a homogeneous gas in equilibrium shows that the results of our experiments are truly universal, realizing the textbook paradigm of a uniform Bose Gas in 2D in thermal equilibrium.

Although the coherent fraction depends on the area from which the light is collected, we show in the Supplementary Materials that the same power law is experimentally observed for different pinhole sizes. The largest pinhole that was used experimentally has a diameter of 12 μm because, for larger pinhole sizes, the assumption of homogeneity breaks down. However, our numerical model allows us to explore the effect of larger pinhole sizes. In agreement with the experiment, our numerical model shows that the effect of the aperture size gives a shifted curve with the same *n*^3.2^ power law (see the Supplementary Materials). Of course, for an infinite system, the coherent fraction goes to zero because an infinite 2D system cannot have long-range order at any finite temperature, but, for any finite area of observation, the same power law will be valid.

The density dependence of properties of a 2D condensate has not been deeply explored in the literature because typical experiments and theory assume a constant density and variation of temperature. We are not aware of any predictions of the observed power law for the coherent fraction, but, because this appears in clearly in both the experiments and simulations for a thermal, homogeneous gas, this should be a universal result. We emphasize the need for further theoretical exploration to give more physical intuition into the origin of the observed power law. It is our hope that our findings will inspire additional theoretical research to understand more deeply this universal power law.

It is quite unexpected that any universal behaviors could be found in a field as well studied for the past 50 years as 2D condensates. This is made possible by the experimental advances of very fine control over the polariton density and long lifetime that allows equilibrium over a wide range of density, as well as the direct in situ measurement of coherence, which is not possible in liquid helium or cold atoms.

## MATERIALS AND METHODS

### Sample design

The microcavities used in this work consisted of a total of 12 GaAs quantum wells with AlAs barriers embedded within a distributed Bragg reflector (DBR). The DBRs are made of alternating layers of AlAs and Al_0.2_Ga_0.8_As. The quantum wells are in groups of 4, with each group placed at one of the three antinodes of the 3λ/2 cavity. The large number of DBR periods gives the cavity a high *Q*-factor, resulting in a cavity lifetime of ∼135 ps and a polariton lifetime of ∼270 ps at resonance. The long cavity lifetime allows polaritons to propagate over macroscopic distances of up to millimeters (*15*). Further details about the samples are discussed in the Supplementary Materials.

## Supplementary Materials

### This PDF file includes:

Sections I to XVI Figs. S1 to S14 References
