# Supplementary material for: Coherence measurements of polaritons in thermal equilibrium reveal a power law for two-dimensional condensates
Source: Sci Adv. 2024 May 3;10(18):eadk6960. doi: 10.1126/sciadv.adk6960 (PMC13108737; doi:10.1126/sciadv.adk6960)
Supplement: Supplementary file 1 — Sections I to XVI Figs. S1 to S14 References [file sciadv.adk6960_sm.pdf]

Science Advances
AAAS

Supplementary Materials for

Coherence measurements of polaritons in thermal equilibrium reveal a power law for two-dimensional condensates

Hassan Alnatah *et al.*

Corresponding author: Hassan Alnatah, haa108@pitt.edu

Sci. Adv. **10**, eadk6960 (2024)
DOI: 10.1126/sciadv.adk6960

This PDF file includes:

Sections I to XVI
Figs. S1 to S14
References

I. SAMPLE DESIGN AND EXPERIMENTAL DETAILS

We have repeated the experiment using two different samples grown at Princeton and at Waterloo, with similar design and cavity Q-factor. The results in the main text are all obtained from the Princeton sample. The two samples have identical design but with different quantum well thicknesses. The Princeton sample has a quantum well thickness of 7 nm, while the Waterloo sample has a quantum well thickness of 8.8 nm. We have designed both sample such that there is a slowly varying cavity gradient allowing us to have multiple detunings across the sample. We chose a location on the Waterloo sample that has the same detuning as the Princeton sample (near resonance) so that a direct comparison can be made. Importantly, the cavity gradient in these samples over short distances is negligible and therefore the polaritons do not feel a force because of this gradient.

The two samples were pumped with different wavelengths since they had different thicknesses, resulting in different wavelength locations for the reflectivity minimum of the cavity. The Princeton sample was pumped with a laser tuned to the second reflectivity minimum (719.5 nm), about 113 meV above the lower polariton resonance. Similarly, the Waterloo sample was pumped with a laser tuned to the second reflectivity minimum (723.7 nm), about 144 meV above the lower polariton resonance.

Since the two samples have a similar strcture and therefore a similar lifetime for the cavity photon, we observed evidence of thermalization in both samples. Typical examples for the occupation number as a function of energy for the Waterloo samples is shown in Fig. S1.

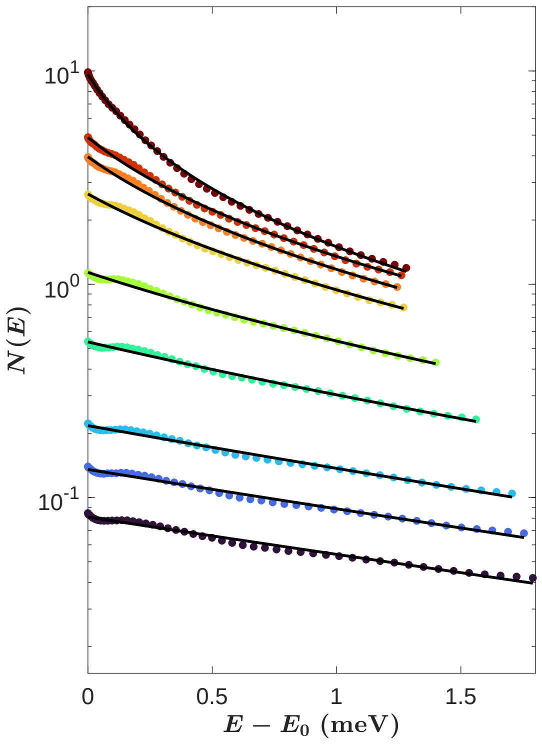

Figure S1: **Equilibrium distribution of polaritons.** Occupation of the lower polariton as a function of energy of the Waterloo sample. The solid lines are best fits to the equilibrium Bose-Einstein distribution.

II. DENSITY CALIBRATION

We used two different methods to calibrate the density of the polaritons and found consistency between them. In this section, we describe the procedure for each method.

In Fig. 5 of the paper, we have used the photon counting method to calibrate the density of the polaritons. To do this, we tuned the laser wavelength to 775 nm to match the polariton emission wavelength. This laser was then sent to a mirror at the sample plane. The mirror at the sample plane reflects the laser beam through the same optical path that was used in the experiment. We then imaged the reflected beam with the CCD camera. Since the total CCD count is proportional to the number of photons, the CCD counts can be written as:

$$I_{\rm CCD} = \frac{1}{\eta}\frac{N_{\rm ph}}{\Delta t}, \tag{S1}$$

where η is the efficiency factor, Δt is the integration time of the camera and $N_{\rm ph}$ is the number of photons detected by the camera. To find the number of photons sent to the camera, we measured the power of the laser. The number of photons that the camera detects during a time Δt is then:

$$N_{\rm ph} = \frac{P\Delta t}{hc/\lambda}, \tag{S2}$$

where P is the measured power of the laser, h is Planck constant, c is the speed of light and λ is the wavelength of the laser. The efficiency factor is then given by:

$$\eta = \frac{P\lambda}{hc}\frac{1}{I_{\rm CCD}}. \tag{S3}$$

Therefore, this allows to relate the number of counts on the camera to the number of photons detected. We used this factor to calibrate the density of the polaritons. The total density of the polaritons for a given $I_{\rm CCD}$ count is then given by:

$$n_{\rm tot} = \frac{\eta I_{\rm CCD}\tau}{A_{\rm obs}}, \tag{S4}$$

where τ is the average radiative lifetime of the polaritons and $A_{\rm obs}$ is the observed area on the sample from which the light was collected. Since the excitation laser beam was chopped in the experiment with a duty cycle d =

1.7%, then the total number of polaritons is

$$n_{\text{tot}} = \frac{\eta I_{\text{CCD}} \tau}{A_{\text{obs}} d}, \tag{S5}$$

where $\tau \approx \tau_{\text{cav}} / |C_{k_\parallel}|^2$. Here $|C_{k_\parallel}|^2$ is the photon fraction and $\tau_{\text{cav}} = 135$ ps [15] is the cavity lifetime. The observed area is given by the pinhole area $A_{\text{obs}} = \pi(6\ \mu\text{m})^2$.

For the second method to calibrate the density of the polaritons, we make use of the fact that our system is in thermal equilibrium. To find the efficiency factor η, we minimized the mean-squared error in fitting a set of distributions $N(E)$ collected at different pump powers to the Bose–Einstein distribution with T and μ as fit parameters for each distribution. This allows us to deduce one single collection efficiency that gives the best fit for distributions simultaneously. This means, for a set of n number of distributions, we have a total of $2n + 1$ free parameter, i.e n temperature parameters, n chemical potential parameters and one single efficiency factor.

We have found consistency between these two methods. At the threshold power $P/P_{\text{th}} = 1$, the photon calibration methods predicts a density of $n = 3.20\ \mu\text{m}^{-2}$ while the best fit to the Bose-Einstein distribution in which both μ and T were allowed to vary predicts a density of $n = 3.12\ \mu\text{m}^{-2}$.

III. DEFINING CRITICAL DENSITY

To determine the critical density threshold of BEC, we measured the total emission intensity for different pump powers. The threshold of condensation is defined from the "S" curve in Fig. S2. Near the condensation threshold, a nonlinear increase in intensity is observed, which becomes linear again at much higher pump power. This nonlinear increase in intensity is what we used to define the threshold of condensation.

The procedure we used to define the power threshold and correspondingly, the density threshold is as follows: We first fitted the data in the linear regime with constant line in a log-log scale. We then defined the threshold at the point when the measured curve deviates from being linear by approximately 10%. Figure S2 shows the fit in the linear regime and the density threshold from the method discussed. The critical density we find is approximately $n_{th} = 3.2\ \mu\text{m}^{-2}$.

The BKT transition density as a function of temperature has been calculated in Ref [21], which is given by

$$n_c = \frac{m k_b T}{2\pi\hbar^2} \ln\left(\frac{\hbar^2 \xi}{mg}\right), \tag{S6}$$

where $\xi = 380$. For a temperature of $T = 20$ K, the calculated BKT transition density of our system is $n_c = 6.3\ \mu\text{m}^{-2}$.

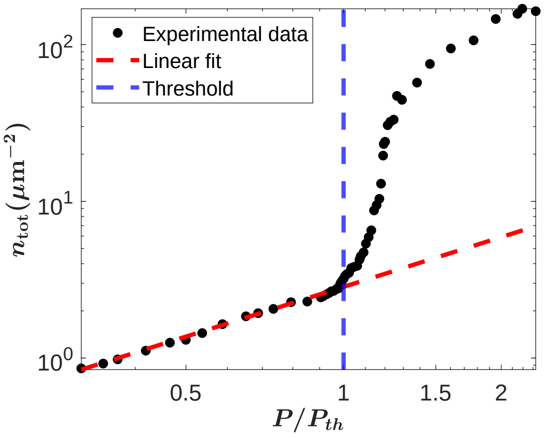

Figure S2: **Condensation threshold.** Polariton density as a function of pump power. The blue vertical line indicates the threshold, which is defined when the the measured curve deviates from being linear by approximately 10%. The threshold density is approximately $n_{th} = 3.2\ \mu\text{m}^{-2}$.

IV. METHOD OF EXTRACTING OCCUPATION NUMBER

We have used angle-resolved imaging to measure the energy resolved emission in k-space $I(k, E)$ of the lower polariton. A typical $I(k, E)$ at low density is shown in Fig. S3(**A**). To extract the occupation number $N(E)$ from the $I(k, E)$ image, we take vertical slices at each k value to obtain $I(E)$ for each k slice. This $I(E)$ curve is then fit with a Lorentzian function to extract the polariton energy for each k slice (see for example Fig. S3(**B**)). The occupation for each k slice is then given by:

$$N(k_i) = C\tau(k_i) \int \mathrm{d}E\ I(k_i, E), \tag{S7}$$

where $\tau(k)$ is the k-dependent radiative lifetime, and C is an overall constant that can be determined from photon counting. The value of C is found by insuring that the total density for the extracted $N(E)$ curve, $\int \mathrm{d}E\ D(E)N(E)$ is equal to the density n_{tot} extracted from photon counting, that is

$$C = \frac{\int \mathrm{d}E\ D(E)N(E)}{n_{\text{tot}}}. \tag{S8}$$

The same C efficiency factor is then used to calibrate the density for each pump power and $D(E) = gm/2\pi\hbar^2$ is the density of states in two dimensions, with $g = 2$ to account for the spin degeneracy.

We note that an alternate approach to find this efficiency factor is by using the second method described in the density calibration section. That is, to treat C as a single efficiency factor that minimizes the mean-squared error in fitting a set of distributions $N(E)$ collected at

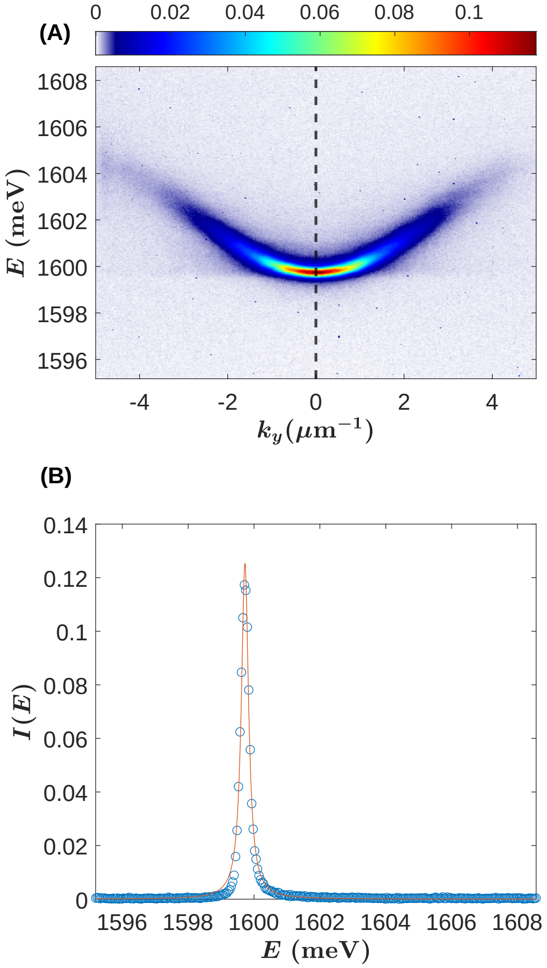

Figure S3: **Polariton occupation extraction method.** **(A)** A typical energy dispersion of the thermalized polariton gas. **(B)** A vertical slice in **(A)** at $k_y = 0$ showing the CCD counts. The red line is a fit to a Lorentzian function predicting an energy 1599.7 meV for the polaritons at $k_y = 0$. The occupation number for this energy is proportional to the area under this Lorentzian curve.

different pump powers to the Bose–Einstein distribution.

V. ENERGY DISPERSION AND EFFECTIVE MASS

Throughout the paper, we have assumed a parabolic dispersion with a fixed effective mass, namely in the density of state calculations and the G-P equation. Accounting for the full polariton dispersion should only provide a small correction, because over the range of wave vectors that we measure, the polariton dispersion is very parabolic (see Fig. S4). The maximum value of wave vector we can measure is determined by the numerical aperture of the microscope objective, which allows a maximum cone of light to exit the lens. Since the polariton dispersion is well approximated by a parabolic dispersion over the experimentally measured wave vector range, we have used a fixed effective mass in the density of states calculations. Non-parabolic effects will only enter for much higher wave vector, i.e. much higher temperature than the experimental conditions.

We note that many-body effects are expected to lead to renormalization of the mass, i.e density dependent mass. Figure S5 shows the effective mass of the polaritons as a function of density. Over the range in which we measure the coherent fraction ($n_{\text{tot}} > 1\ \mu\text{m}^{-2}$), we find that the mass remains mostly constant as the density is increased. We cannot reliably extract the mass for densities larger than in Fig. S5. This is because at very high density, the condensate at $k = 0$ becomes too bright and the occupation of states $k \neq 0$ are too dim that they are comparable to the noise in our measurements.

VI. ENERGY LINEWIDTH

In addition to extracting the occupation number from the fitting procedure described in the previous section, we have also extracted the energy linewidth $I(E)$ at $k = 0$ for each pump power. The linewidth is extracted by fitting $I(E)$ at $k = 0$ with a Lorentzian as shown in Fig. S3**(B)** for each pump power. In this case, it is more helpful to plot the linewdith as a function of the occupation at ground state energy rather than the total polariton density. As shown in Fig. S6, the linewidth stays constant at low density and then increases due to collisional processes as the density increases. However, when the polartion gas becomes degenerate (i.e, $N(E = 0) \sim 1$), significant line narrowing is observed, which is a hallmark of Bose-Einstein condensation.

VII. AVERAGE KIENTIC ENERGY

From the T and μ fits shown in Fig. 3 of the main text, we have calculated the average energy per particle,

$$\bar{E} = \frac{\int \mathrm{d}E\ ED(E)N(E,T,\mu)}{\int \mathrm{d}E\ D(E)N(E,T,\mu)} = \frac{\int \mathrm{d}E\ EN(E,T,\mu)}{\int \mathrm{d}E\ N(E,T,\mu)}, \tag{S9}$$

where the density-of-states factor cancels out because the density of states is independent of energy in two dimensions. For each T and μ values extracted from the fits, we plot the average energy per particle as a function of the total polariton density, as shown in Fig. S7**(A)**. Initially, the average energy per particle increases, presumably due

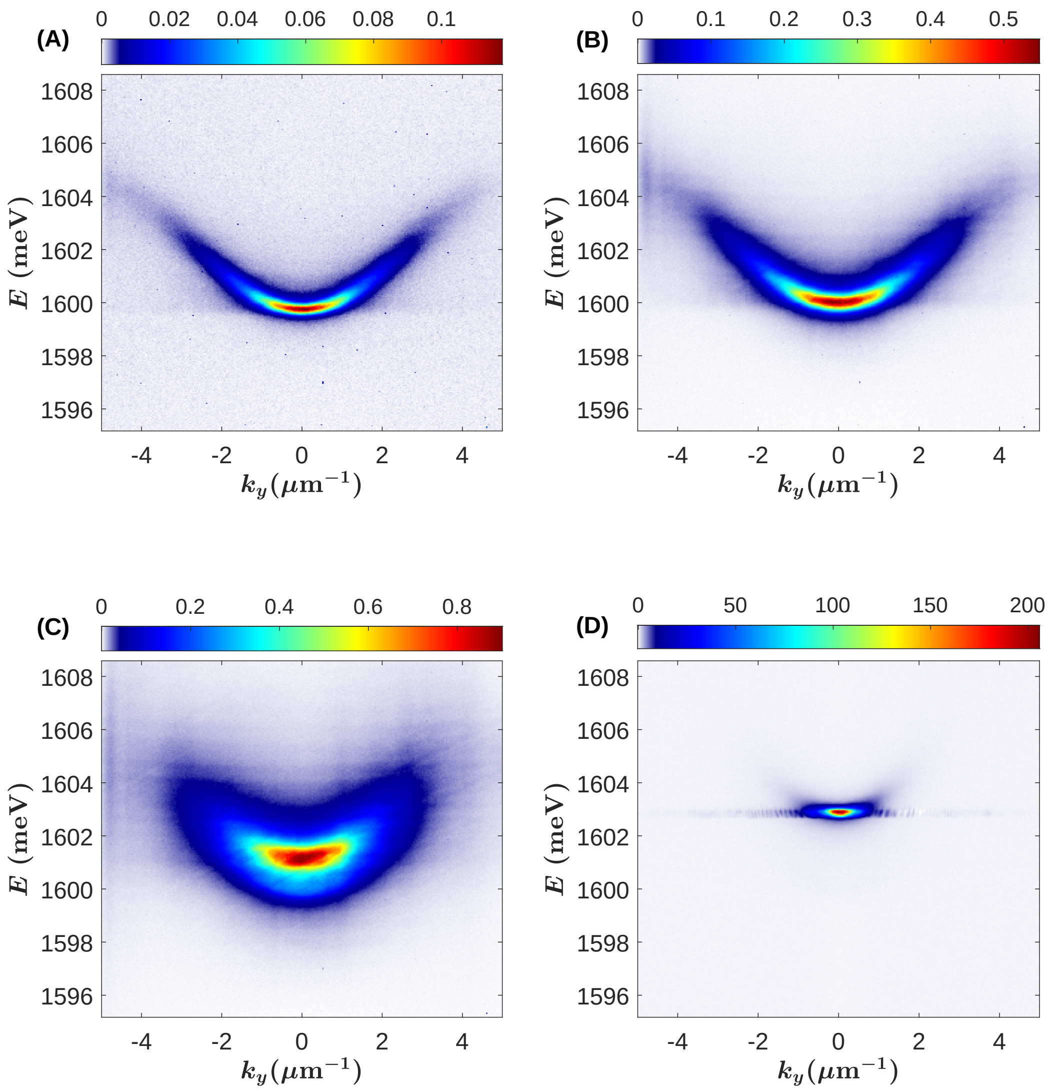

Figure S4: **Energy dispersion of the lower polariton.** Typical energy dispersion images of the thermalized polariton gas at **(A)** 0.008 P/P_{th}, **(B)** 0.132 P/P_{th}, **(C)** 0.530 P/P_{th} and **(D)** 1.265 P/P_{th}

to heating of the lattice due to the excess energy of the pump laser, seen also in the fit temperatures of Figure 3**(A)** of the main text. However, when the polariton gas becomes degenerate, the average energy per particle decreases significantly.

We take this decrease as primarily due to the fact that a degenerate Bose gas has lower average energy as the density increases, at constant temperature. As a comparison, we calculate the average energy per particle expected for a Bose gas at a constant temperature (Fig. S7**(B)**). Since the occupation becomes peaked at $E = 0$ at high density, the average energy per particle has to decrease when the polariton gas becomes degenerate, which is in qualitative agreement with the experiment.

VIII. INHOMOGENEITY AT HIGH DENSITY

Although the polariton gas is fairly homogeneous for a large range of density within the observed area, at very high density we see evidence of self-trapping into the central region of the pump. To characterize the homogeneity of the polariton gas, we plot the full width at 90% of the maximum of the polariton density extracted from the real space images as shown in Fig. S8**(A)**. As a reference, we show at which density the polariton gas has a full width

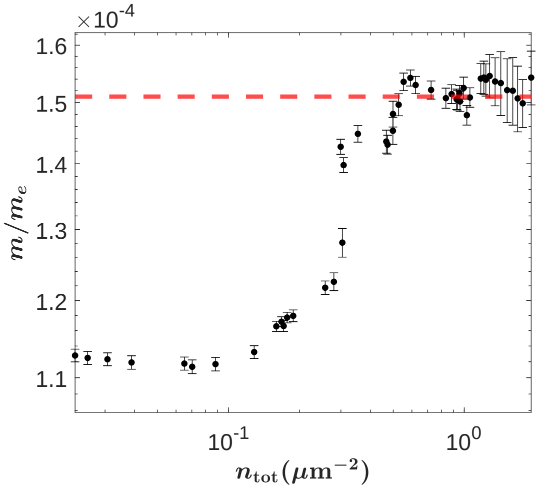

Figure S5: **Polariton effective mass.** The extracted lower polariton effective mass as a function of density. The dashed line is the mass used in the simulations.

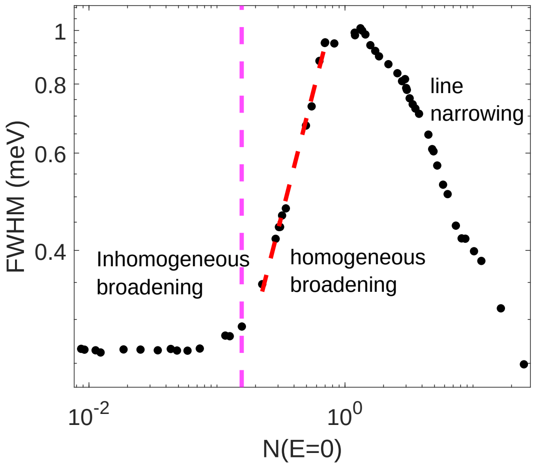

Figure S6: **Linewidth narrowing and broadening.** Energy linewidth at $k = 0$ as a function of the ground state occupation. Red line: power law of 1.0 indicating the regime of homogeneous broadening. Magenta line: a horizontal line indicating the regime of inhomogeneous broadening. Blue line: The crossing regime between inhomogeneous broadening and homogeneous broadening. When the occupation becomes comparable to 1, the linewidth decreases sharply due to linewidth narrowing near the onest of condensation.

at 90% of the maximum equal to the diameter of the pinhole in the experiment. This is shown in the $n_0/n_{\rm tot}$ plot in Fig. S8(**B**) for the case of a pinhole with a radius $r = 6\ \mu$m.

As seen in Fig. S8(**B**), the gas is fairly homogeneous for more than three orders of magnitude of the value of the coherent fraction. However, at very high density, the full width at 90% maximum of the gas becomes smaller

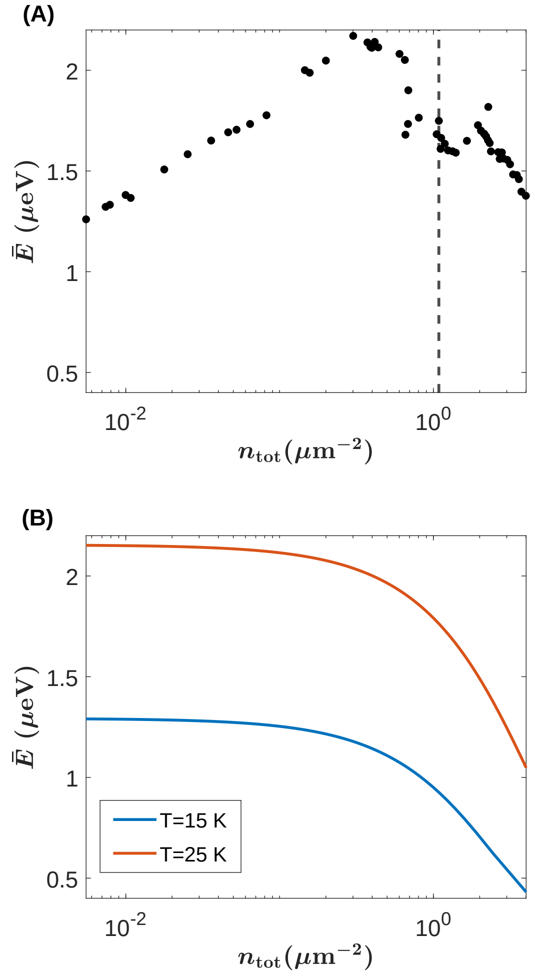

Figure S7: **Average energy per particle.** (**A**) The experimental average energy per particle calculated from Eq. (S9) (**B**) The average energy per particle calculated using the Bose-Einstein distribution for a constant temperature $T = 15$ K and $T = 25$ K.

than the size of the pinhole leading to inhomogeneity of the polarion gas.

IX. POLARIZATION MEASUREMENTS

We have measured the polarization of the polariton gas for different pump powers by using a half wave plate. The half wave plate was rotated in small angle steps and the intensity was recorded for each angle. This allows us to plot the polarization of the polariton gas for different densities as shown in Fig. S9(**B-I**). The polariton gas remains unpolarized in the power law regime and becomes polarized at very high density. Initially, the polarization direction becomes pinned to the gradient direction

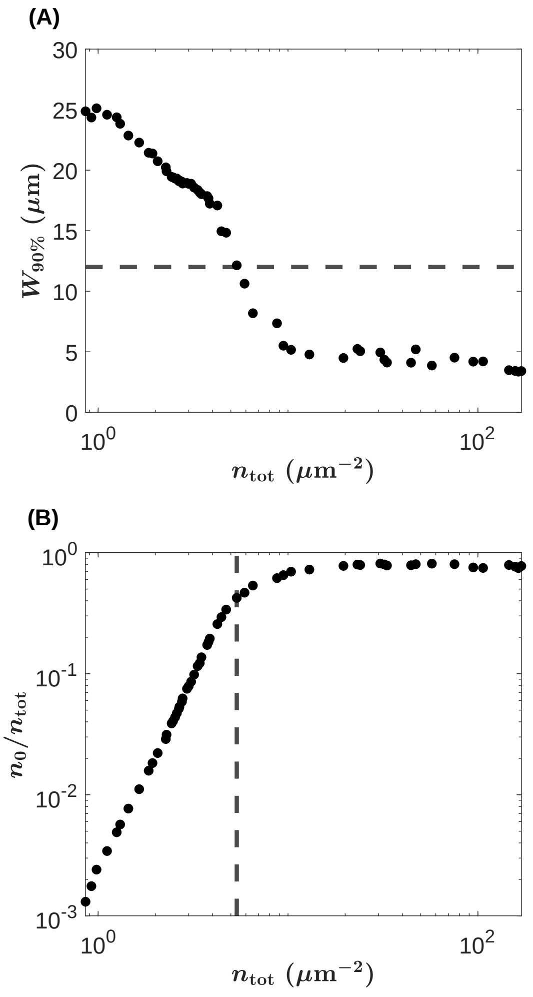

Figure S8: **Homogeneity of the polariton gas. (A)** Full width at 90% maximum of the polariton density in real space as a function of the total integrated density. The dashed line indicates the diameter of the pinhole used in the main text. **(B)** The coherent fraction as a function of the total polariton density, for the same data set. The dashed line is the density at which the full width at 90% maximum of the polariton profile becomes equal to the size of the pinhole.

($\theta = 0°$) of the cavity as shown in Fig. S9**(E-F)**. At very high density, the polarization is pinned to the [110] crystalline axis, which corresponds to $\theta = 15°$ in polarization plot (Fig. S9**(B-I)**.)

X. METHOD OF EXTRACTING THE COHERENT FRACTION

As discussed in the main text, we have use the interference images in k-space to extract the coherent fraction. These interference images were then fit to the function,

$$I(k) = N(k)\left[1 + \alpha e^{-k/\kappa}\cos(\lambda k)\right], \tag{S10}$$

where κ is a fit parameter giving the region of coherence, α is a fit parameter ranging between 0 and 1 giving the degree of coherence, and λ is the component associated with the fringe spacing. For $N(k)$, we have used a Gaussian function of the form $N(k) = Be^{-k^2/\sigma_k^2}$, where B and σ_k are fit parameters. Therefore, in total we have four different parameters to fit κ, α, B and σ_k. The fringe spacing parameter λ can be extracted from the data directly.

We have used the same method to extract the coherent fraction for the experiment and the simulations. First, we take a slice of the interference pattern $k_y = 0$, which we then fit to the model mentioned above. Figure S10 shows an example of the interference pattern $I(k_y = 0, k_x)$ for the experiment and the resulting fit to Eq. (S10). From this, we extracted the coherent fraction by computing the integral,

$$\frac{n_0}{n_{tot}} = \frac{\alpha \int \mathrm{d}^2k\, N(k)e^{-k/\kappa}}{\int \mathrm{d}^2k\, N(k)}. \tag{S11}$$

For example, for the interference patten in (Fig. S10**(B)**), we obtained the following parameters for the best fit, $\kappa = 0.92$ μm, $\alpha = 0.96$, $B = 0.49$ and $\sigma_k = 0.34$ μm. This then gives a coherent fraction $n_0/n_{\text{tot}} = 0.70$. This procedure is repeated for each density, allowing us to plot the coherent fraction as a function of the polariton density. In Fig. S10**(A)**, we include a typical fit to the interference pattern below the threshold.

XI. TEMPORAL COHERENCE MEASUREMENTS

In addition to measuring the coherence in k-space, we have also measured the coherence time of the polariton gas. By introducing a time delay between the two interferometer arms, we have measured the time correlation function $g^{(1)}(\Delta t)$. This is done by computing the integral of the visibility, that is n_0/n_{tot}, using the procedure described in the previous section (Eq. (S11)) for each time delay as shown in Fig. S11**(A)**. To extract the coherence time, we have fitted the visibility function with a Gaussian (Fig. S11**(A)**), allowing us to extract the coherence time for each density. The coherence time is defined as the FWHM of the fitted Gaussian. Figure S11**(B)** shows the coherence time as a function of the polarion density. The value of about 2 ps at the lowest density is consistent with the line broadening reported at that density in previous work, which was attributed to decoherence due to polariton-exciton collisions [22].

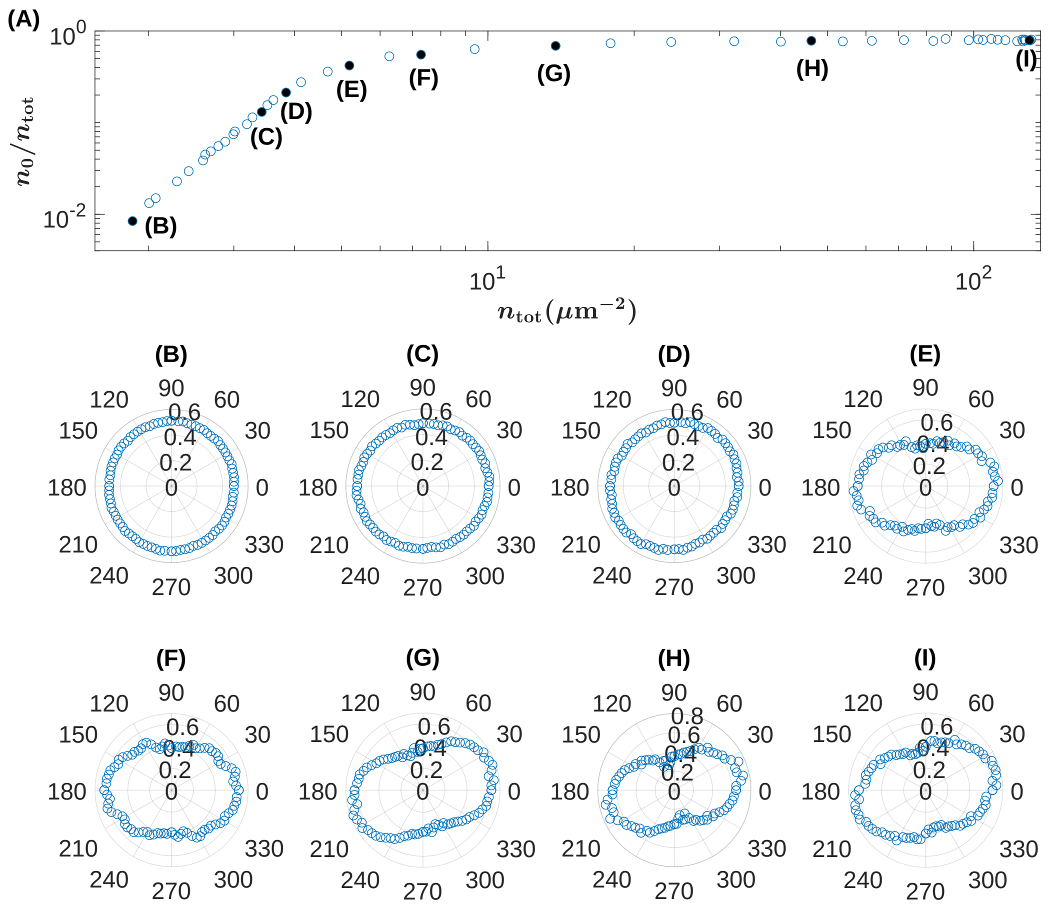

Figure S9: **Polarization of the polariton gas.** **(A)** Experimentally measured coherent fraction as a function of the total polariton density for a pinhole with an area $A = \pi(6\ \mu\text{m})^2$ **(B-I)** The intensity of the polariton gas as a function of the polarization angle in degrees. The points at which the polarization data are taken are labeled in **(A)** by the black solid circles. The polarization of the polariton gas at high density is mostly pinned to the [110] crystalline axis, which corresponds to 15°. The gradient of the cavity is alighned along the horizontal direction.

XII. EFFECT OF CHANGING PINHOLE SIZE

We have found that the same power law for the coherent fraction is seen for different pinhole sizes from which the light is collected in real space. The largest pinhole that was used experimentally has a radius of 6 μm since for larger pinhole sizes, the assumption of homogeneity breaks down (see Fig S8**(A)**). Figure S12**(A)** shows a comparison of the experimentally measured coherent fraction for three examples of different pinhole sizes. The same power law is observed for these different cases.

In agreement with the experiment, our numerical model shows that the effect of the aperture size gives a shifted curve with the same power law. Figure S12**(B)** shows five different examples, starting from a radius of $r = 3\ \mu$m and increase the radius by a factor of $\sqrt{2}$, which corresponds to increasing the area by a factor of two. Although the largest area we can explore experimentally is $\pi(6\ \mu\text{m})^2$, our numerical analysis shows that this power law is still maintained even after increasing the area by a factor of four.

XIII. REAL-SPACE CORRELATION FUNCTION

In addition to calculating the coherent fraction, we have also calculated the correlation length in our numerics. For a two-dimensional system, the correlation function can be written as

$$g^{(1)}(\Delta r) = \frac{\langle \psi^*(r+\Delta r,t)\,\psi(r,t)\rangle}{\sqrt{\left\langle |\psi(r+\Delta r,t)|^2\right\rangle \left\langle |\psi(r,t)|^2\right\rangle}}. \qquad \text{(S12)}$$

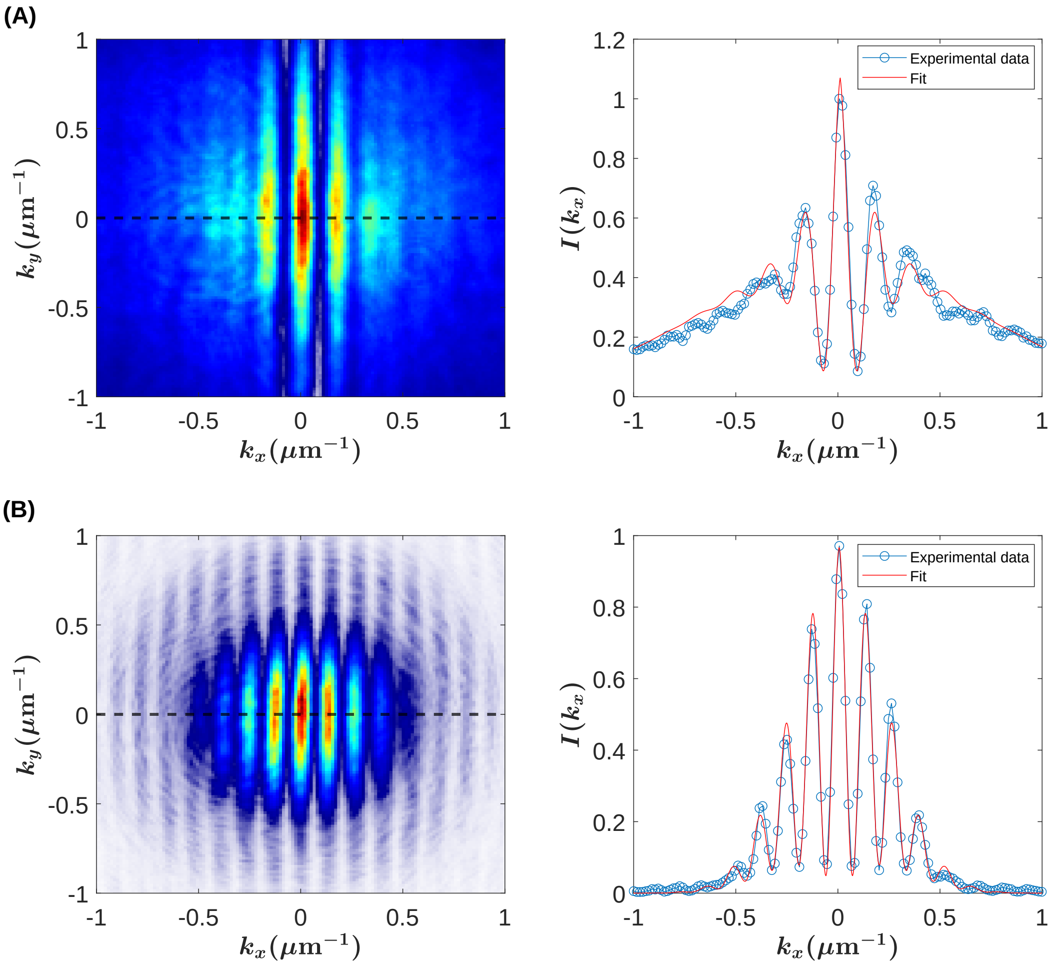

Figure S10: **Low and high density examples of the best fit to Eq. (S10). (A, left panel)** A typical experimental interference image in k-space below the threshold. **(A, right panel)** horizontal slice in **(A, left panel)** at $k_y = 0$ as indicated by the dashed line. **(B, left panel)** A typical experimental interference image in k-space above the threshold. **(B, right panel)** horizontal slice in **(B, left panel)** at $k_y = 0$ as indicated by the dashed line. The red line is the best fit to Eq. (S10)

The correlation function is calculated at a sufficiently late time after transients have died down. Figure S13**(A)** shows the correlation function calculated for different densities. A crossover from exponential to algebraic decay of the first order correlations is clearly observed, which is a characteristic feature of the BKT transition.

XIV. CORRELATION AREA

In this section, we make a connection between the coherent fraction and the correlation area. The interference pattern we measure in k-space can be written as:

$$\begin{aligned} I(\vec{k}) &= \left|\psi\left(\vec{k}\right) + \psi\left(-\vec{k}\right)\right|^2 \\ &= \left|\psi\left(\vec{k}\right)\right|^2 + \left|\psi\left(-\vec{k}\right)\right|^2 + 2\,\mathrm{Re}\left[\psi^*\left(\vec{k}\right)\psi\left(-\vec{k}\right)\right]. \end{aligned} \tag{S13}$$

Assuming $\left|\psi\left(\vec{k}\right)\right|^2 = \left|\psi\left(-\vec{k}\right)\right|^2 = N(k)$ gives,

$$I(\vec{k}) = 2N(k) + 2\,\mathrm{Re}\left[\psi^*\left(\vec{k}\right)\psi\left(-\vec{k}\right)\right]. \tag{S14}$$

One, therefore, can define a coherent fraction $\Delta = n_0/n_{\mathrm{tot}}$ as:

$$\Delta = \frac{\int \mathrm{d}^2k\ \psi^*(\vec{k})\psi(-\vec{k})}{\int \mathrm{d}^2k\ \left|\psi(\vec{k})\right|^2}, \tag{S15}$$

where the wavefunction in k-space is given by the Fourier transform of $\psi(\vec{r})$.

$$\psi(\vec{k}) = \frac{1}{2\pi}\int \mathrm{d}^2r\ \psi(\vec{r})e^{i\vec{k}\cdot\vec{r}}. \tag{S16}$$

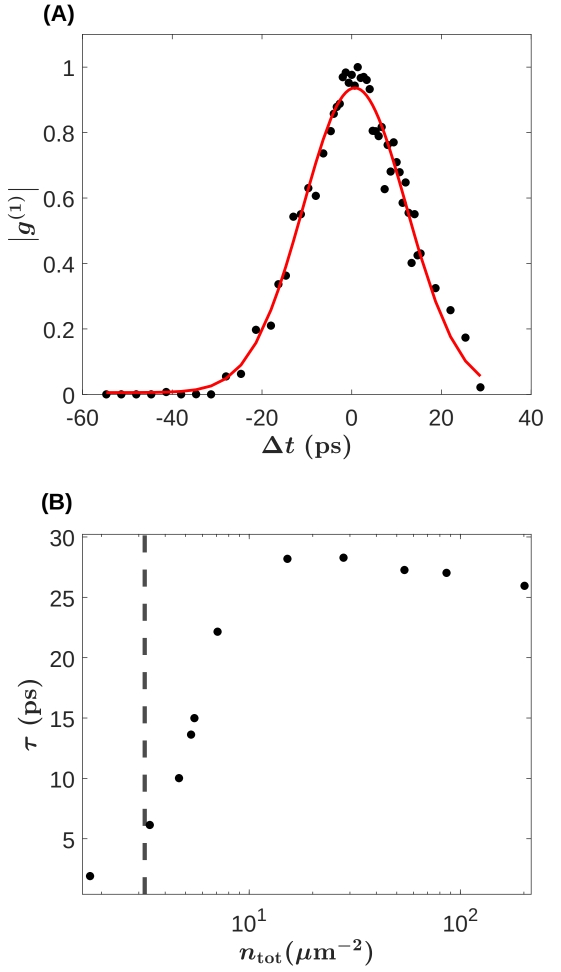

Figure S11: **Coherence time**. **(A)** A typical visibility curve as a function of the time delay between the two Michelson arms. The red line is a Gaussian fit used to extract the coherence time. **(B)** Coherence time as a function of density measured by varying the arm of the Michelson interferometer. The vertical line indicates the critical density.

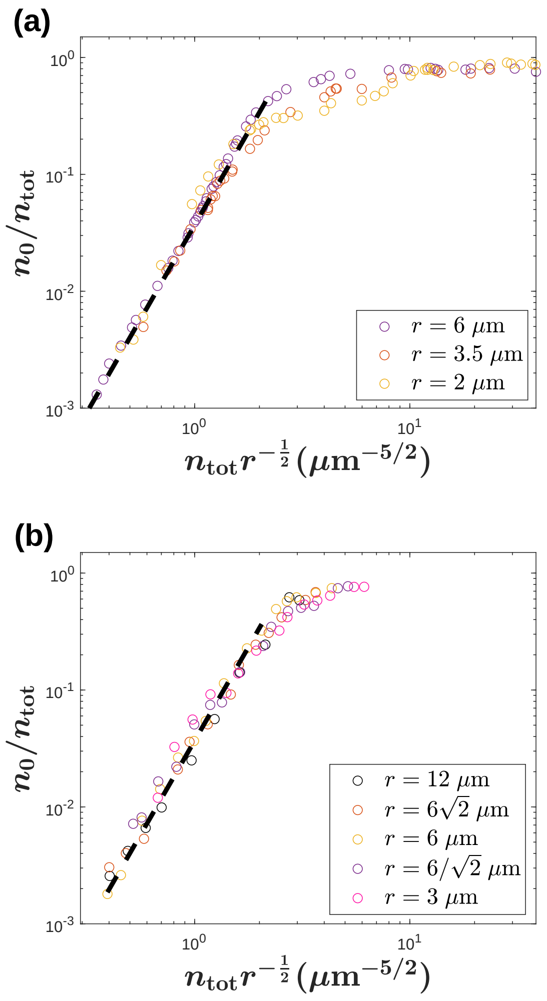

Figure S12: **Effect of different pinhole sizes.** **(A)** The experimentally measured coherent fraction for three different pinhole sizes collapsed onto a single curve. **(B)** The condensate fraction extracted from the numerics for five different pinhole sizes collapsed onto a single curve. The dashed line is a power law of 3.2.

Plugging in Eq. S16 into Eq. S15 and using the relation $\int \mathrm{d}^2k\, e^{-i\vec{k}\cdot(\vec{r}+\vec{r}')} = 2\pi\delta\left(\vec{r}+\vec{r}'\right)$, we obtain

$$\Delta = \frac{\int \mathrm{d}^2r\, \psi^*(\vec{r})\psi(-\vec{r})}{\int \mathrm{d}^2r\, |\psi(\vec{r})|^2}. \qquad \text{(S17)}$$

The origin $\vec{r} = 0$ is defined arbitrarily here. In a translationally invariant system, we should average over all origins $\vec{r}$:

$$\begin{aligned}\Delta &= \frac{1}{n_{\text{tot}}A}\int \mathrm{d}^2r\, \langle\psi^*(\vec{r})\psi(-\vec{r})\rangle \\ &= \frac{1}{n_{\text{tot}}A^2}\int \mathrm{d}^2r' \int \mathrm{d}^2r\, \psi^*(\vec{r'}+\vec{r})\psi(\vec{r'}-\vec{r}) \qquad \text{(S18)} \\ &= \frac{1}{n_{\text{tot}}A^2}\int \mathrm{d}^2r \int \mathrm{d}^2r''\, \psi^*(\vec{r''}+2\vec{r})\psi(\vec{r''}),\end{aligned}$$

where $\vec{r''} = \vec{r'} - \vec{r}$, A is the area and n_{tot} is the total density. Using the definition of the correlation function

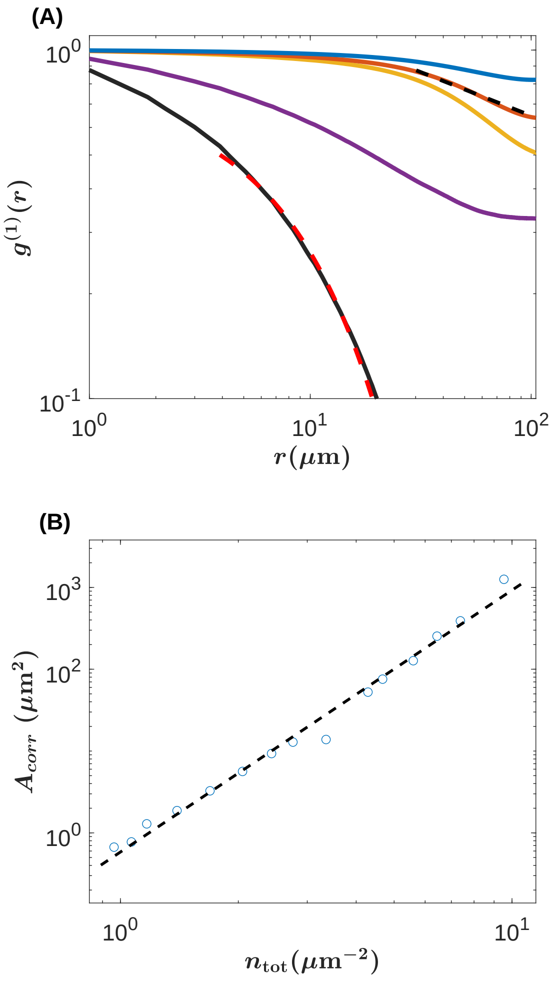

Figure S13: **Correlation function and correlation area. (A)** The correlation function calculated for density values from small to high, 7.29 μm^{-2}, 11.71 μm^{-2}, 14.97 μm^{-2}, 15.39 μm^{-2} and 16.11 μm^{-2}. Red dashed line: fit to exponential decay. Black dashed line is a $r^{-1/4}$ power law, which is expected at the BKT transition density. **(B)** The correlation area minus the zero-density correlation area, as a function of the total density. The black dashed line is a power law of 3.2.

in Eq. S12, we have:

$$\Delta = \frac{1}{A}\int \mathrm{d}^2r\, g^{(1)}(2r) \equiv \frac{A_{\mathrm{corr}}}{A}, \tag{S19}$$

where A_{corr} is the correlation area, which is given by

$$A_{\mathrm{corr}} = \int \mathrm{d}^2r\, g(2r). \tag{S20}$$

Therefore, in the low density limit, the coherent fraction is proportional to the correlation area $\Delta \sim A_{\mathrm{corr}}$. The correlation length is related to the correlation area via the relation

$$l_{\mathrm{corr}} = \sqrt{\frac{A_{\mathrm{corr}}}{\pi}} \sim \Delta^{\frac{1}{2}}. \tag{S21}$$

Figure S13**(B)** shows the correlation area calculated from our model as a function of density. The correlation area is fit by a power law of $n^{3.17\pm0.21}$. (Similar to the condensate fraction calculations, we subtract the zero-density limit of the correlation area, which corresponds to the Maxwell-Boltzmann limit.) This power law is consistent with the experimental measurements and with the calculated coherent fraction from the numerics since $A_{\mathrm{corr}} \sim \Delta \sim n^{3.2}$. The power law is still maintained in the numerics even for the full system size of $L = 210\ \mu$m, which is much larger than the thermal de Broglie wavelength of the polaritons $L >> \lambda_{th} = h/\sqrt{2\pi m k_B T} \sim 1\ \mu$m.

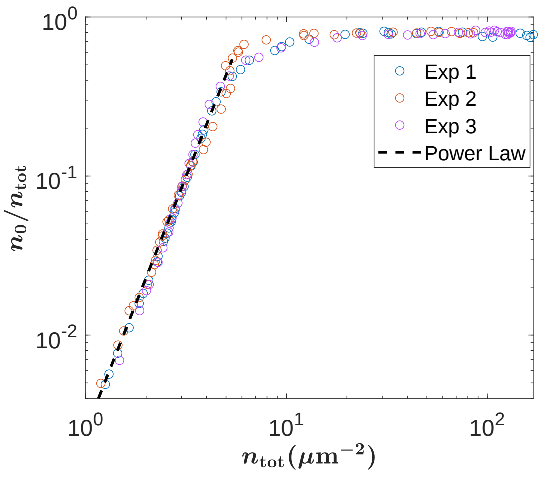

Figure S14: **Multiple data sets for the coherent fraction.** Different data sets showing the same power law for a pinhole with an area $A = \pi(6\ \mu\mathrm{m})^2$. Exp 1 is the results obtained from the Princeton sample. Exp 2 is obtained from the same sample on a different day and a different location on the sample. Exp 3 is obtained from the Waterloo sample. The dashed line is a power law of 3.2.

XV. COMPARISON OF DIFFERENT DATA SETS

We have found consistent results for different data sets that were obtained during different experiments. We have also repeated the experiment using two different samples grown at Princeton and at Waterloo, with similar design and cavity Q-factor (i.e., comparable polariton lifetime) and have found consistent results, in particular, the same power law for the quasicondensate fraction. Figure S14 compares multiple data sets.

XVI. NUMERICAL METHOD

As mentioned in the main text, we solved the Gross-Pitaevskii equation with noise introduced in the initial conditions,

$$i\hbar\frac{\partial\psi(\mathbf{r},t)}{\partial t} = \left[-\frac{\hbar^2\nabla^2}{2m} + g_c|\psi(\mathbf{r},t)|^2\right]\psi(\mathbf{r},\mathbf{t}). \tag{S22}$$

The Gross-Pitaevskii is solved numerically adopting a Runge-Kutta method of order fourth on a two-dimensional numerical grid with $N = 512^2$ points with grid-spacing $a = 0.41$ μm. We have used the following experimental parameters in the fit shown: $m = 1.515 \times 10^{-4} m_e$ with m_e the electron mass, and $g = 2.105$ $\mu\text{eV} - \mu\text{m}^2$ for the interaction strength. Because the correlation of the coherence depends sensitively on gn, where n is the density, and we know the absolute density of the polaritons from the good fits to the Bose-Einstein distribution, as discussed in the main text, these experiments are an independent measurement of the polariton-polariton interaction constant. Accounting for the uncertainty in the density calibration, we find $g = 2.11 \pm 0.25$ $\mu\text{eV} - \mu\text{m}^2$. Since the polaritons are approximately 55% excitonic in these experiments, this corresponds to a polariton-exciton interaction constant $\tilde{g} = 3.83$ $\mu\text{eV} - \mu\text{m}^2$, or an exciton-exciton interaction constant of 6.96 $\mu\text{eV} - \mu\text{m}^2$. The number found here is below some previously reported experimental values, but well above the theoretical prediction of $g \sim 0.25$ $\mu\text{eV} - \mu\text{m}^2$ [22].

To avoid reflections from the boundaries, we have used periodic boundary conditions. We have ensured that system size is sufficiently larger than the experimental size of the system to avoid any boundary effects influencing the relevant region. The numerical results presented in the main text are for a system size of 210 μm.

We have assumed that the system is initially given by an incoherent equilibrium state,

$$\psi(x,y,t=0) = \sum_{k_n}\sum_{k_m}\sqrt{N\left(\sqrt{k_n^2+k_m^2}\right)}\,e^{i(k_n x + k_m y)} \times e^{i(\theta_{k_n}+\theta_{k_m})}, \tag{S23}$$

where $N(k)$ is given by the Bose-Einstein distribution,

$$N(k) = \frac{1}{e^{(E(k)-\mu)/k_B T} - 1}. \tag{S24}$$

We have fixed the temperature to be $T = 20$ K in the numerics and varied the chemical potential μ. Therefore, the choise of μ defines the density of the system, which is given by,

$$n_{\text{tot}} = \int \mathrm{d}E\, D(E) N(E,T,\mu) \tag{S25}$$

where $D(E)$ is the constant density of states in two-dimensions. For each chemical potential value, the wavefunction was evolved in time for $t_{\text{max}} = 1$ ns. The interference pattern $I(k_x,k_y)$ was then calculated using the equation described in the main text and then averaged over 20 independent stochastic paths each with different random initial conditions for θ_{k_x} and θ_{k_y}.

REFERENCES

1. N. D. Mermin, H. Wagner, Absence of ferromagnetism or antiferromagnetism in one- or two-dimensional isotropic Heisenberg models. *Phys. Rev. Lett.* **17**, 1133–1136 (1966).
2. V. Berezinskii, Destruction of long range order in one-dimensional and two-dimensional systems having a continuous symmetry group. I. Classical systems. *Sov. Phys. JETP* **32**, 493–500 (1971).
3. J. M. Kosterlitz, D. J. Thouless, Ordering, metastability and phase transitions in two-dimensional systems. *J. Phys. C: Solid State Phys.* **6**, 1181–1203 (1973).
4. T. Plisson, B. Allard, M. Holzmann, G. Salomon, A. Aspect, P. Bouyer, T. Bourdel, Coherence properties of a two-dimensional trapped Bose gas around the superfluid transition. *Phys. Rev. A* **84**, 061606 (2011).
5. Z. Hadzibabic, P. Krüger, M. Cheneau, B. Battelier, J. Dalibard, Berezinskii–Kosterlitz–Thouless crossover in a trapped atomic gas. *Nature* **441**, 1118–1121 (2006).
6. P. Clade, C. Ryu, A. Ramanathan, K. Helmerson, W. D. Phillips, Observation of a 2D Bose gas: From thermal to quasicondensate to superfluid. *Phys. Rev. Lett.* **102**, 170401 (2009).
7. N. Navon, R. P. Smith, Z. Hadzibabic, Quantum gases in optical boxes. *Nat. Phys.* **17**, 1334–1341 (2021).
8. H. Deng, G. Weihs, C. Santori, J. Bloch, Y. Yamamoto, Condensation of semiconductor microcavity exciton polaritons. *Science* **298**, 199 –202 (2002).
9. J. Kasprzak, M. Richard, S. Kundermann, A. Baas, P. Jeambrun, J. M. J. Keeling, F. M. Marchetti, M. H. Szymańska, R. André, J. L. Staehli, V. Savona, P. B. Littlewood, B. Deveaud, L. S. Dang, Bose–Einstein condensation of exciton polaritons. *Nature* **443**, 409–414 (2006).
10. R. Balili, V. Hartwell, D. Snoke, L. Pfeiffer, K. West, Bose-Einstein condensation of microcavity polaritons in a trap. *Science* **316**, 1007 –1010 (2007).
11. M. Abbarchi, A. Amo, V. G. Sala, D. D. Solnyshkov, H. Flayac, L. Ferrier, I. Sagnes, E. Galopin, A. Lemaître, G. Malpuech, J. Bloch, Macroscopic quantum self-trapping and Josephson oscillations of exciton polaritons. *Nat. Phys.* **9**, 275–279 (2013).
12. D. Sanvitto, F. M. Marchetti, M. H. Szymańska, G. Tosi, M. Baudisch, F. P. Laussy, D. N. Krizhanovskii, M. S. Skolnick, L. Marrucci, A. Lemaître, J. Bloch, C. Tejedor, L. Viña, Persistent currents and quantized vortices in a polariton superfluid. *Nat. Phys.* **6**, 527–533 (2010).
13. K. Lagoudakis, T. Ostatnick'y, A. Kavokin, Y. G. Rubo, R. André, B. Deveaud-Plédran, Observation of half-quantum vortices in an exciton-polariton condensate. *Science* **326**, 974–976 (2009).
14. B. Nelsen, G. Liu, M. Steger, D. W. Snoke, R. Balili, K. West, L. Pfeiffer, Dissipationless flow and sharp threshold of a polariton condensate with long lifetime. *Phys. Rev. X* **3**, 041015 (2013).
15. M. Steger, C. Gautham, D. W. Snoke, L. Pfeiffer, K. West, Slow reflection and two-photon generation of microcavity exciton–polaritons. *Optica* **2**, 1 (2015).
16. Y. Sun, P. Wen, Y. Yoon, G. Liu, M. Steger, L. N. Pfeiffer, K. West, D. W. Snoke, K. A. Nelson, Bose-Einstein condensation of long-lifetime polaritons in thermal equilibrium. *Phys. Rev. Lett.* **118**, 016602 (2017).
17. D. Caputo, D. Ballarini, G. Dagvadorj, C. S. Muñoz, M. De Giorgi, L. Dominici, K. West, L. N. Pfeiffer, G. Gigli, F. P. Laussy, M. H. Szymańska, D. Sanvitto, Topological order and thermal equilibrium in polariton condensates. *Nat. Mater.* **17**, 145–151 (2018).

18. L. Chomaz, L. Corman, T. Bienaimé, R. Desbuquois, C. Weitenberg, S. Nascimbene, J. Beugnon, J. Dalibard, Emergence of coherence via transverse condensation in a uniform quasi-two-dimensional Bose gas. *Nat. Commun.* **6**, 6162 (2015).
19. G. Malpuech, Y. Rubo, F. Laussy, P. Bigenwald, A. Kavokin, Polariton laser: Thermodynamics and quantum kinetic theory. *Semicond. Sci. Technol.* **18**, S395–S404 (2003).
20. T. Doan, H. T. Cao, D. T. Thoai, H. Haug, Condensation kinetics of microcavity polaritons with scattering by phonons and polaritons. *Phys. Rev. B* **72**, 085301 (2005).
21. N. Prokof'ev, B. Svistunov, Two-dimensional weakly interacting Bose gas in the fluctuation region. *Phys. Rev. A* **66**, 043608 (2002).
22. D. Snoke, V. Hartwell, J. Beaumariage, S. Mukherjee, Y. Yoon, D. Myers, M. Steger, Z. Sun, K. Nelson, L. Pfeiffer, Reanalysis of experimental determinations of polariton-polariton interactions in microcavities. *Phys. Rev. B* **107**, 165302 (2023).
